# FieldTrip: Open Source Software for Advanced Analysis of MEG, EEG, and Invasive Electrophysiological Data

**DOI:** 10.1155/2011/156869

**Published:** 2010-12-23

**Authors:** Robert Oostenveld, Pascal Fries, Eric Maris, Jan-Mathijs Schoffelen

**Affiliations:** ^1^Donders Institute for Brain, Cognition and Behaviour, Centre for Cognitive Neuroimaging, Radboud University Nijmegen, 6500 HB Nijmegen, The Netherlands; ^2^Ernst Strüngmann Institute and Max Planck Society, D-60528 Frankfurt, Germany

## Abstract

This paper describes FieldTrip, an open source software package that we developed for the analysis of MEG, EEG, and other electrophysiological data. The software is implemented as a MATLAB toolbox and includes a complete set of consistent and user-friendly high-level functions that allow experimental neuroscientists to analyze experimental data. It includes algorithms for simple and advanced analysis, such as time-frequency analysis using multitapers, source reconstruction using dipoles, distributed sources and beamformers, connectivity analysis, and nonparametric statistical permutation tests at the channel and source level. The implementation as toolbox allows the user to perform elaborate and structured analyses of large data sets using the MATLAB command line and batch scripting. Furthermore, users and developers can easily extend the functionality and implement new algorithms. The modular design facilitates the reuse in other software packages.

## 1. General Overview

FieldTrip is a MATLAB-toolbox for the analysis of MEG, EEG, and other electrophysiological data, which is freely available from http://www.ru.nl/neuroimaging/fieldtrip under the GNU public license. The development of FieldTrip started in 2003 at the F.C. Donders Centre for Cognitive Neuroimaging and up to today it continues to be actively developed at the Donders Institute for Brain, Cognition and Behaviour of the Radboud University Nijmegen, the Netherlands, together with collaborating researchers and institutes.

The software is fully implemented in MATLAB, a high-level technical computing language and interactive environment for algorithm development, data analysis, and visualization, which is available for all commonly used computer platforms (http://www.mathworks.com). MATLAB is widely known and used in the neuroimaging community. Although MATLAB is relatively expensive, the investment is easily compensated by the rich feature set and flexibility it provides.

**Figure 1:**
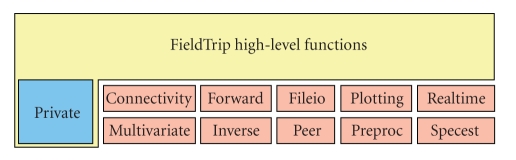
The structure of the toolbox.

The FieldTrip toolbox consists of approximately 108 high-level and 858 low-level functions with in total 103227 lines of code. The main focus is on the analysis of noninvasive and invasive electrophysiological data, including spike recordings, but in theory any time series data (e.g., BOLD or NIRS time courses) can be analysed. The toolbox supports reading data from a large number of different file formats (Table 1). Supported functionality includes algorithms for data preprocessing, event-related field/response analysis, parametric and nonparametric spectral analysis, forward and inverse source modelling, connectivity analysis, classification, real-time data processing, and statistical inference. Finally, the toolbox contains a module allowing for peer-to-peer distributed computing. The structure of the toolbox with its modules is shown schematically in Figure 1.

An important goal of the FieldTrip project is to provide a common platform for experimental scientists and methods developers. The FieldTrip toolbox allows experimental scientists to have access to state-of-the-art data analysis algorithms. For methods developers it facilitates their algorithms to be applied to a large variety of experimental data.

The organization of the FieldTrip project facilitates a highly dynamic development model with a rapid availability of software updates to the user. This is realized by a daily release of the latest version on an FTP-server. Next to this, the documentation is fully available online as a wiki (http://www.ru.nl/neuroimaging/fieldtrip), which promotes active contributions of both users and methods developers. The FieldTrip wiki contains a large amount of documentation for facilitating the use of the toolbox, including tutorial documentation, answers to frequently asked questions and example MATLAB code. Finally, there is an active e-mail discussion list, with approximately 650 subscribers (state of August 2010).

The focus of this paper is on features that discriminate FieldTrip from other (publicly available) toolboxes as described elsewhere in this issue. We will first describe FieldTrip from the user's perspective, followed by a description from the developer's perspective. Both sections highlight important features relevant for the specific target group. Subsequently, specific features of the different modules are summarized. This paper ends with some concluding remarks on the FieldTrip project as a whole.

## 2. The User Perspective

### 2.1. No Graphical User Interface

An important feature of the FieldTrip toolbox is that it does not have a Graphical User Interface (GUI). Instead, the user is interacting directly with the functions on the MATLAB command line or in scripts. Consequently, users need to have some basic knowledge of MATLAB in order to fruitfully use the toolbox. Although this requires an initial investment from the side of the user, it allows for very flexible combination of the functions to suit specific analysis needs.

The FieldTrip toolbox consists of high- and low-level functions. The high-level functions provide a consistent and easy-to-use interface of the functionality to the users, enabling them to do the analysis in well-defined steps. The low-level functions implement the core functionality, but are not designed to be used by the common neuroscience researcher and do not provide an easy programming structure to implement a complete analysis of the data. The low-level functions are largely hidden from the regular end users in private directories.

### 2.2. Analysis Scripts to Mix and Match

Practically, users start by writing an analysis script, in which they mix and match the FieldTrip high-level functions according to the experimental research question. A script consists of a sequence of FieldTrip function calls, each of which performing a specific part of the analysis pipeline. If required, the users extend the analyses with their own code. The content and style of analysis scripts highly depend on the expertise and programming skills of the user. In general the resulting scripts can be thought of as (parts of) analysis protocols. The scripts can be easily used for batch processing, allowing for a convenient application of the same analysis protocol to multiple subjects or experiments. Also, scripts can be exchanged between users, and between students and their supervisors, facilitating collaboration and knowledge transfer.

### 2.3. A Typical FieldTrip Function Call

High-level FieldTrip functions have a well-defined function-call interface. The input to a particular FieldTrip function consists of one or more MATLAB structures: a configuration structure, optionally followed by one or more data structures. The input configuration structure contains the options or parameters that specify how the data will be processed by the function and/or how the algorithm will behave in detail. The input data structure is usually the output of a FieldTrip function that was called earlier in the analysis pipeline (see below).

### 2.4. Configuration Structure

The specification of the parameters in the configuration structure follows the user's perspective: channels are for example indicated with their label and physical quantities are expressed in SI-units (e.g., frequency in Hz). Configuration parameters are stored in fields that express their meaning in human readable names. If possible, default values will be assigned to parameters that have not been specified by the end user.

### 2.5. Output of the FieldTrip Function

The output of a FieldTrip function is a MATLAB structure containing the processed data. This data structure also includes the configuration field that was used for the computations inside the function, allowing the user to inspect the details of the analysis, for example, the default configuration settings that were used. Some FieldTrip functions do not produce output data, but rather a figure displaying the data. A small set of FieldTrip functions generates neither data, nor figures, but extends the input configuration structure.

### 2.6. Definition of Data Structures

**Figure 2:**
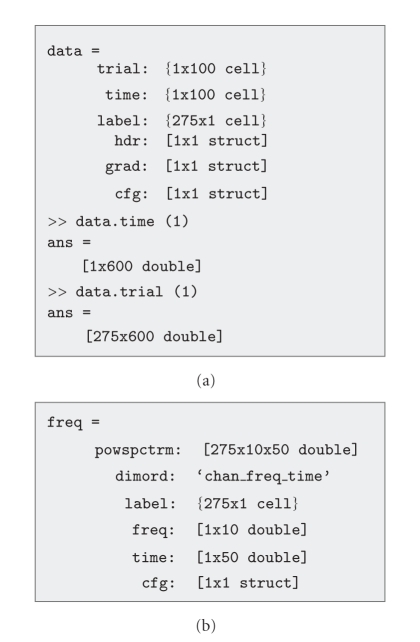
Data representation examples. (a) Epoched time domain, sensor-level data. (b) Time-frequency representation of sensor-level data.

FieldTrip makes use of a number of well-defined data structures which are designed to be parsimonious, yet complete. They contain the numeric representation of the data in combination with the information necessary to interpret this numeric data. There are certain types of data structures for the different representations of the data. For example, segmented sensor-level time domain data is stored in a structure of data type “raw”. Structures of this data type consist of a cell-array “trial”, in which each cell contains a Channels × Timepoints matrix, a cell-array “label”, referring to the label of each of the channels, and a cell-array “time”, in which each cell contains the 1 × Timepoints vector, providing temporal information for each of the samples in each of the trials (Figure 2(a)).  Figure 2(b) shows an example of a structure of data type “freq”.

### 2.7. Analysis Scripts for Step-by-Step Analysis Are Protocollike

**Figure 3:**
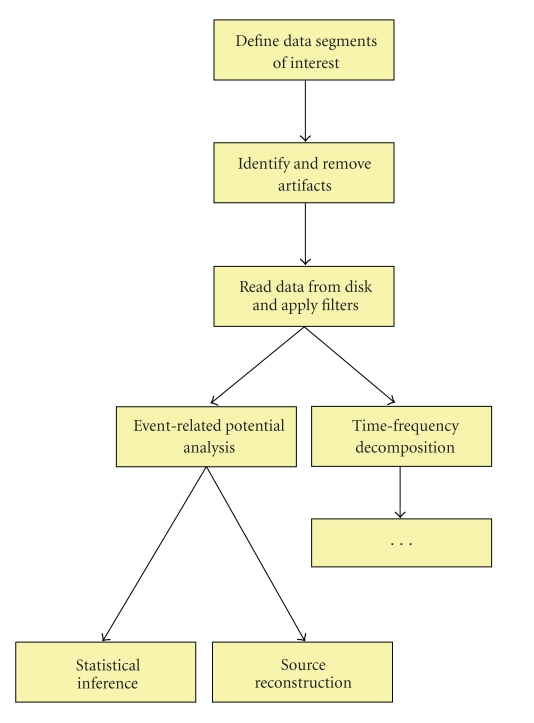
Example analysis pipeline.

**Figure 4:**
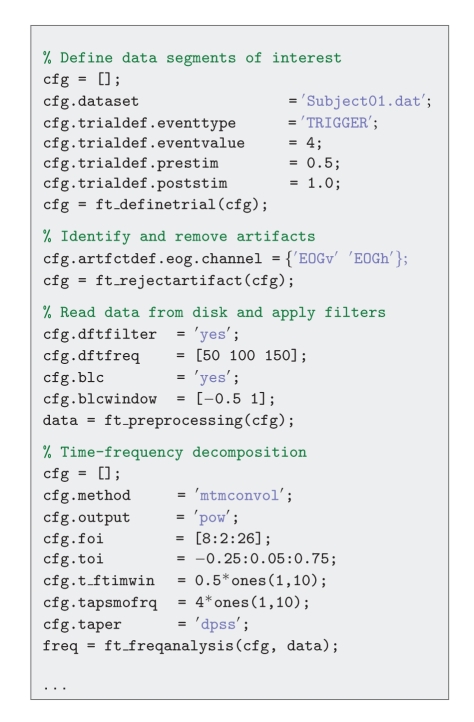
Example analysis script.

As mentioned before, analysis scripts usually contain a sequence of FieldTrip function calls. Each analysis step is usually performed by a single high-level FieldTrip function. To illustrate this, the following paragraphs and Figure 3 describe an analysis pipeline, showing the one-to-one mapping between a conceptual analysis step, and a high-level FieldTrip function.  Figure 4 gives an impression of the corresponding analysis script.

#### 2.7.1. Define Data Segments of Interest

A typical analysis starts with reading and segmenting the data such that the experimental conditions are represented as trials in a data structure. For simple experimental designs, segmenting the data can be done using a standard function that is included. For complex experimental designs, the user can provide his or her own function that decodes the sequence of triggers. Specific to FieldTrip is the possibility to create and analyze segments of variable length. One can think of segmenting the data as inverting the implementation of the experimental design in the stimulus presentation software. The definition of the boundaries of the relevant data segments is generated by ft_definetrial.

#### 2.7.2. Identify and Remove Artifacts

Once the interesting segments of data have been identified, one may want to identify artifacts in the data that would affect the quality of the analysis results. Subsequently, the user can either remove the affected segments from the data altogether, or remove the artifact from the data by applying a linear projection.

**Figure 5:**
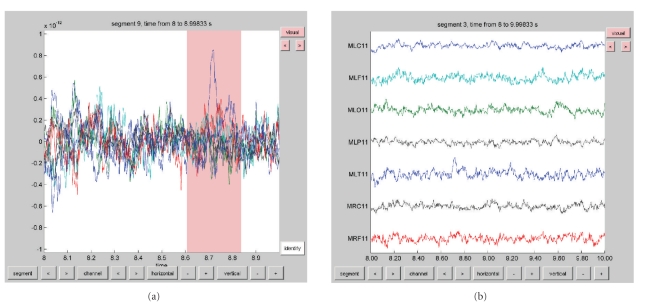
Visualization of data with the ft_databrowser function. (a) Display of a set of sensors in the “butterfly” mode, showing the possibility to select segments of interest, for example, to identify artifacts (pink box). (b) Display of a set of sensors in the “vertical” mode.

The function ft_rejectartifact allows for semiautomatic detection of well-defined artifacts such as eye blinks, muscle contractions, or MEG SQUID jumps. With a minimum of user interaction artifacts are identified by thresholding the data after processing the data to increase the sensitivity to pick up the characteristics of the specific artifact. For example, MEG SQUID jumps are easily detected after applying a median filter to the data. Alternatively, users can use the ft_databrowser function, allowing them to browse through the data and manually identify data segments containing artifacts (Figure 5). 

To project out artifacts with a characteristic spatial topography, such as eye blinks or cardiac activity, the ft_componentanalysis function can be used. This implements a variety of blind source separation methods, such as independent component analysis (ICA, based on code from the EEGLAB toolbox [1]), and principal component analysis (PCA).

#### 2.7.3. Read Data from Disk and Apply Filters

The ft_preprocessing function is used to read the interesting segments of data from disk into the MATLAB workspace and to apply various processing steps to the raw data such as filtering, rereferencing and baseline correction. The input to ft_preprocessing is a single configuration structure, specifying the filename of the raw data file, as well as the definition of the segments of interest. In addition, the configuration contains the instructions for the various optional processing steps.

#### 2.7.4. Event-Related Potential Analysis

Once the segmented data are in the MATLAB workspace, the next step could be to average across trials of a particular experimental condition to obtain the event-related field (ERF) using the function ft_timelockanalysis.  Figure 7 gives some examples of visualizing the ERFs.

#### 2.7.5. Time-Frequency Decomposition

Alternative to the analysis of event-related fields, the experimental question may warrant the data to be analysed in the frequency domain. The transformation from the time domain into the frequency domain is achieved by ft_freqanalysis.

#### 2.7.6. Source Reconstruction

**Figure 6:**
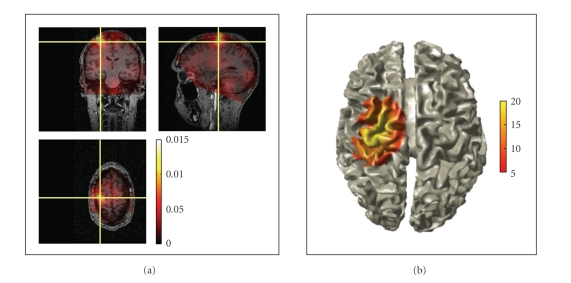
Visualization of source-reconstructed data. (a) Three-dimensional orthographic rendering of corticomuscular coherence with opacity mapping. (b) Surface rendering of statistically thresholded corticomuscular coherence after Z-transformation.

Reconstructing the location and the strength of the underlying neuronal activity can either be done with ft_sourceanalysis or ft_dipolefitting. The latter function implements a nonlinear optimization algorithm that fits a prespecified number of dipoles to the data [2]. The ft_sourceanalysis function implements distributed source models, such as minimum norm estimates (MNE) [3], and beamformers for time-domain and frequency-domain data [4, 5]. The source space can be defined either as a three-dimensional regular grid, or can be based on a triangulation of the cortical sheet. There is no functionality in FieldTrip for the creation of cortical meshes, since excellent and freely available toolboxes such as FreeSurfer [6] already exist. Distributed source data and beamformer maps as well as statistically transformed derivations from these maps can be readily visualized in combination with anatomical information using the ft_sourceplot function (Figure 6). Functional data can be statistically thresholded and plotted on top of the anatomy, using opacity mapping. Threedimensional volumetric data can be rendered onto a template or individual cortical mesh (Figure 6(b)).

#### 2.7.7. Statistical Inference

Usually the final step in a particular analysis stream is the assessment of statistical significance of the observed experimental effect, either at the level of a single subject or across subjects. At this point in the analysis, the data can be expressed in the time domain, frequency domain, or time-frequency domain. Furthermore, the data can either be represented at the sensor or at the source level. Independent of the data representation, FieldTrip uses the same underlying code to assess significance using parametric or nonparametric algorithms [7] for statistical inference.

### 2.8. Handling the Data

An important concept in FieldTrip is that the data are in the hands of the end users. The data flows through the different FieldTrip functions and is transformed along the way. After each transformation the data corresponds to a variable, that is, a data structure in the MATLAB workspace, and the user can optionally save it to disk. The data serving as the input to a particular FieldTrip function is not included in its output. The user has to explicitly manage the data, that is, assign meaningful variable names and save the variables to disk as a MATLAB mat file, especially if intermediate analysis results need to be revisited. In addition to saving workspace variables to mat files, analysis results can be exported to a number of non-MATLAB file formats that are supported by external software. Sensor-level electrophysiological data can, for example, be saved in EDF and BrainVision Analyzer format, source reconstructed volumetric data can be saved in NIfTI format.

Although the data at the different levels of the analysis are not kept within a single structure, the history of the analysis is still present at any stage. Each output data structure of a FieldTrip function contains a nested configuration field. This field not only holds the parameters used to generate the data at the present level, but also contains the parameters used to process the data at all previous levels. In this way, information about the processing history is present at any level of the analysis pipeline.

### 2.9. Batch Processing

For most cognitive experiments the data from many subjects is required. Recent technological developments allow for recordings with more channels, leading to larger data sets. Therefore, despite advancements in computational power, the analysis of MEG/EEG data remains computationally demanding and takes a significant amount of time. To analyze experiments with a large number of subjects, batch processing is convenient and often necessary to systematically explore the outcome of the analysis given a particular set of parameters.

One of the preferred ways of using batch processing in a FieldTrip analysis is to start with the construction of a single script containing the full analysis pipeline for a single subject. During the implementation of this script, the user extends the script in the MATLAB editor and uses copy-and-paste to execute segments of the script. Once the user is satisfied with the sequence of analysis steps and with the parameter settings, this script can be converted into batch analysis. This can be implemented by breaking the single script into separate components, each of which resulting in an intermediate result that the user wants to inspect and/or save to disk. By adding a for loop around each of these components, the whole analysis pipeline can easily be executed for all subjects. Parameters that are specific to the individual subjects can be put in an additional subject-specific script, which is evaluated inside the batch component scripts. The whole batch can be easily reevaluated with different parameter settings.

### 2.10. Visualizing Analysis Results

MATLAB contains a variety of high-quality and multipurpose plotting tools and visualization of (intermediate) analysis results is often possible using these standard MATLAB plotting functions. Important for this is that the numeric representation of the data is easily accessible in the FieldTrip datastructures. However, complex analysis results and the multidimensional nature of some data representations sometimes prohibit easy visualization or exploration. To this end FieldTrip contains several high-level plotting functions for channel and source level data representations. For example ft_multiplorTFR allows the user to visualize the spatiotemporal spectral data and to interactively explore all three dimensions by making selections of channels and along the time and/or frequency axis. 

**Figure 7:**
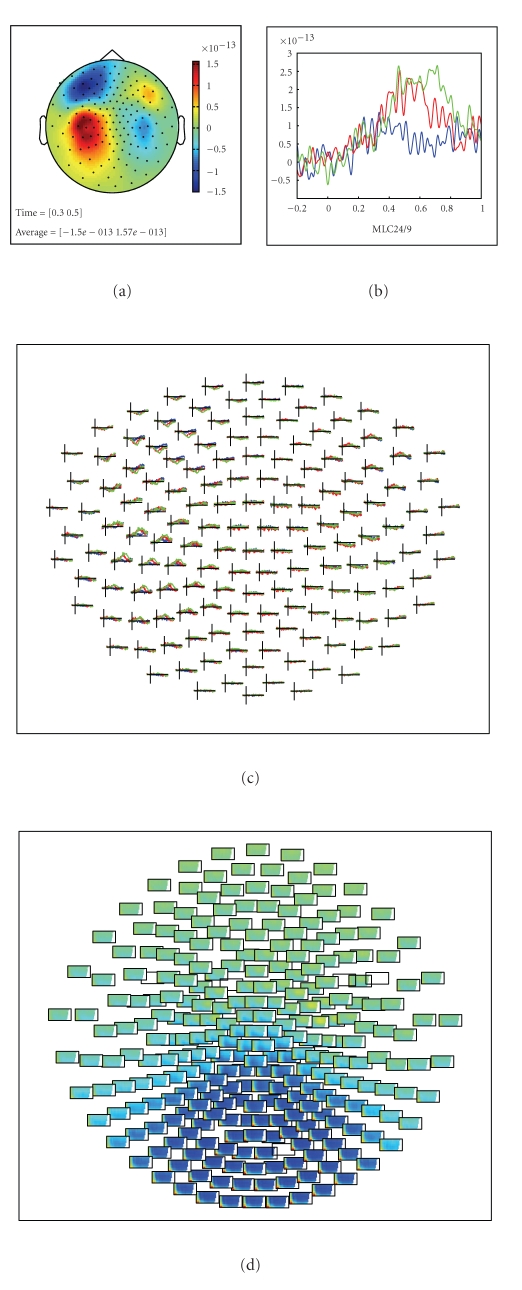
Visualization of multidimensional data. (a) Topographical representation of a specific temporal component of the ERF. (b) Single sensor display of an ERF. (c) Topographical display of sensor-level ERFs in three experimental conditions. (d) Topographical display of sensor-level TRFs.

Examples of the graphical output of some plotting functions are shown in Figures 6 and 7. Sensor-level data can be visualized by interpolating it on a two-dimensional projection of the sensor positions, for example, to look at the spatial distribution of specific ERF components (Figure 7(a)), or the specific oscillatory components in the time-frequency representation (TFR) of the data. Another method to visualize sensor-level data is to plot the complete ERF or TFR at each sensor position (Figures 7(c) and 7(d)) or to plot the averaged ERF or TFR over a subset of channels (Figure 7(b)). Source-reconstructed data can be visualized in combination with anatomical information using the ft_sourceplot function using orthogonal MRI slices or a 3D surface rendering of the cortical sheet (Figure 6). Relevant for exploring the data is the interactive option, enabling the user to select with the mouse regions-of-interest in time, frequency, and/or space. Finally, the ft_databrowser (Figure 5) can be used to interactively explore time-domain sensor-level data, at the same time allowing for the visually guided specification of artifacts.

## 3. The Developer's Perspective

Contrary to other software where the GUI provides the central structure for the end user and, consequently, in which the developer has to adhere to the GUI structure, FieldTrip is specifically targeted at being a toolbox rather than an application. The functions in the toolbox are implemented at a level that allows them to be used in (batch) scripts, but at the same time to be called from other MATLAB-based software.

Working in a high profile scientific setting requires the experimental scientists to keep up with the latest methodological developments. Therefore, the distinction between user and developer is often not so clearcut. As already mentioned, the FieldTrip project aims at providing a common platform for end users and for methods developers, but also tries to be useful for researchers in between.

FieldTrip started to be developed in the context of a young and rapidly growing neuroimaging centre. As a consequence, the researchers involved were constantly pushing for improved and extended functionality. This led to a development model that is still in use to date. Characteristic is the continuously evolving codebase as opposed to fixed-point releases. The users rely on the latest daily released version. The developers take great care in ensuring continuity by providing backward compatibility in the many, but small, steps that the codebase takes. This is facilitated by the separation of FieldTrip into high-level functions with a stable function-call interface and well-defined data structures, and low-level functions that can easily be modified and extended to meet the evolving requirements.

### 3.1. Contributing Code to FieldTrip

In general, methods developers may want to contribute code to FieldTrip because it offers a unique opportunity to get innovative analysis methods applied to a large variety of real-world experimental data. Furthermore, contributing new methods results in feedback from the user community, which can result in new insights into the methods themselves.

For a methods developer it is easy to add a new high-level function, because of the parsimonious data representation, and because of the one-to-one relation between analysis steps and single FieldTrip functions. By utilizing an existing FieldTrip data structure as input to the function, and by remaining close to another data structure as output format for the function, the method developer does not have to be concerned with specific data formats and subsequent processing and visualisation of the analysis results. By combining the separate functions, the FieldTrip user has an exponential increase in possible combinations of functionality.

### 3.2. Using FieldTrip Code Elsewhere

During the last two years the code has been modularised to clarify the layered organisation of some of the low-level functionality. Having a well-defined structure for the modules simplifies the maintenance of the code. Furthermore, it facilitates the collaboration with methods developers and with developers of other software. Each module contains intermediate- and low-level functions related to a particular type of computation, for example, the forward module contains functions for the computation of lead fields, and the fileio module contains functions for reading in raw data of various file formats. The function-call interface for the intermediate-level module functions works with key-value pairs to specify the behaviour, rather than with a configuration structure. Compared to the use of a single configuration structure with parameters in the high-level FieldTrip functions, key-value pairs represent a more widely used programming style.

The modular design facilitates reuse of source code in other software. For example, SPM8 and FieldTrip share the same fileio module, which is clearly beneficial for both the end users and the code developers: code does not need to be written twice, and both the SPM and FieldTrip users can access a comprehensive set of data formats. Apart from sharing the fileio, forward and preproc modules, SPM uses FieldTrip for various MEG and EEG analysis algorithms, whereas FieldTrip uses SPM for processing anatomical MRI data for the purpose of spatial normalisation and segmentation. Besides the active collaboration with the SPM developers, FieldTrip shares code with other noncommercial and commercial software such as EEGLAB, BESA, BCI2000 and others.

## 4. Specific Details Related to the Different Modules

The following part provides some additional details related to the functionality of the different modules, without the aim of being exhaustive. 

### 4.1. Fileio

The fileio module implements a consistent interface to electrophysiological sensor level data from many acquisition systems by separating the information in the datasets into header information, events (such as triggers), and the actual recorded signals. The intermediate-level reading functions perform file format detection and automatically select the appropriate low-level reading functions. The different file formats supported by this module are shown in Table 1.

### 4.2. Preproc

The preproc module contains algorithms for time domain filtering, rereferencing, baseline correction, detrending, and other functions that are usually associated with the preprocessing of raw data.

### 4.3. Specest

Nonparametric (Fourier transform based) and parametric spectral analysis methods are implemented in the specest module. It contains algorithms for estimating the power and/or phase of oscillatory components using (time-) frequency decomposition, wavelets, and multitapers [8].

### 4.4. Connectivity

This module provides functionality to compute measures of bivariate and multivariate connectivity. Originally, FieldTrip evolved from code that was written to analyze sensor and source-level coherence in MEG. Past years have witnessed an increased interest in studying connectivity, which has led to the emergence of open source toolboxes specifically designed for this purpose [9]. The FieldTrip connectivity module focuses on the analysis of connectivity in the frequency domain. Various connectivity metrics are available, such as coherence, phase locking value [10], imaginary part of coherency [11], phase slope index [12], partial directed coherence [13], directed transfer function [14], and Geweke's extension of Granger causality to the frequency domain [15].

### 4.5. Forward

This module contains functions to compute lead fields, that is, the solutions to the forward problem. Various algorithms are implemented, including for MEG single sphere [16], overlapping spheres [17], and a spherical harmonics approximation of realistic geometries [18]. For EEG, single and multiple concentric sphere models and the boundary element method (BEM) are available [19, 20].

### 4.6. Inverse

Different source reconstruction algorithms are available for the estimation of the location and strength of neuronal activity, including dipole fitting based on nonlinear optimization, [2] scanning methods such as minimum variance beamformers in the time and frequency domain [4, 5], and linear estimation of distributed source models [3].

### 4.7. Multivariate

The multivariate module contains a wide range of machine learning algorithms for classification, such as linear discriminant analysis, support vector machines, Bayesian networks, Gaussian mixture models, and groupwise logistic regression [21]. The classification algorithms can be used for offline single trial analysis, and for online brain-computer interface (BCI) applications.

### 4.8. Plotting

This module contains intermediate-level functions that facilitate the visualization of complex data such as multichannel time-frequency decompositions or source reconstructions.

### 4.9. Realtime

Although MATLAB itself is a largely single-threaded application that provides an interpreted programming environment, it is highly suited for rapid application development and is sufficiently fast for real-time analysis of multichannel EEG and MEG data. The core functionality of the real-time module is provided by the FieldTrip buffer, a multithreaded network transparent TCP server that allows the acquisition client to stream data in small blocks, while at the same time allowing analysis of the data in MATLAB. This module allows the user to build BCI systems.

### 4.10. Peer

Efficient use of available computation resources speeds up the often time-consuming analysis of electrophysiological data. The peer module boasts a zero configuration, dynamically adjusting peer-to-peer network that allows for sharing computational resources among multiple users. It allows the user to distribute multiple computational jobs in parallel over multiple MATLAB sessions running on a single computer, or, just as easily, running on different computers in the network.

## 5. Concluding Remarks

The features of the FieldTrip software that set it apart from the other free and commercially available EEG/MEG analysis software are that it allows for analyzing experimental designs in which the trial duration varies, the elaborate implementation of spectral analysis using multitapers, statistical inference using nonparametric permutation tests and source reconstruction with beamformer methods. There is an active involvement of the users through the e-mail discussion list and online wiki documentation system.

The open source development model of FieldTrip has proven to be very effective, on the one hand creating a large and well-tested collection of MATLAB functions, on the other hand resulting in a large contribution to experimental neuroscience. The latter is exemplified both by the large number of publications in which FieldTrip is used (>100) and by the high impact factor of those publications, among others in Science, Nature, Neuron, Current Biology, PNAS, and the Journal of Neuroscience. The open nature of the FieldTrip project has resulted in a community with an active exchange of scientific ideas between users and developers.

Besides the impact that FieldTrip itself has on experimental neuroscience, just as important is the contribution of the open source model to scientific research. There is a healthy competition between different EEG/MEG software packages, which results in an ongoing drive for improved methods and improved usability of the software. The open source development model fits very well with the scientific approach of providing the information required for obtaining reproducible findings. Sharing the source code pushes forward both the fields of neuroscience methods and experimental neuroscience.

When we embarked on our journey to create, use, and share new ideas for the analysis of electrophysiological data, we did not yet realise the full potential of FieldTrip. Looking back over the short, but intense history of the project, the most rewarding are the scientific and personal fulfilment resulting from the interaction with all the great researchers that we got to know through this project.

## Figures and Tables

**Table 1:** File formats supported by FieldTrip. Less common file formats are excluded from this listing but can be found on the website.

Class of data	Manufacturer/file format
MEG file formats	CTF/VSM
	Neuromag/Elekta
	BTi/4D Neuroimaging
	Yokogawa/KIT
	Chieti ITAB system

EEG file formats	BrainProducts/BrainVision
	NeuroScan
	Electrical Geodesics, Inc.
	Megis software/BESA research
	Biosemi
	BCI2000
	ANT/EEProbe
	Curry
	Micromed
	Nexstim
	European data format
	Generic standard formats

Anatomical MRI formats	Dicom
	NIfTI
	Analyze
	MINC
	AFNI

	Neuralynx
Animal electrophysiology	Plexon
file formats	Tucker Davis Technology
	Cambridge Electronic Design
